# AF-CuRL: Stable Reinforcement Learning for Resource-Constrained Long-Form Reasoning in Edge-Intelligent Systems

**DOI:** 10.3390/s26051433

**Published:** 2026-02-25

**Authors:** Ziqin Yan, Yurong Wang, Qingsheng Yue, Xiaojiang Wang

**Affiliations:** 1 School of Electronic and Information Engineering, Lanzhou Jiaotong University, Lanzhou 730070, China; 13240096@stu.lzjtu.edu.cn; 2 Cloud Gansu Technology Co., Ltd., Lanzhou 730030, China; muliwyr@163.com (Y.W.); xhwxjor@163.com (X.W.); 3 School of Statistics, Renmin University of China, Beijing 100872, China; 4 School of Physics and Electronic Engineering, Northwest Normal University, Lanzhou 730070, China

**Keywords:** large language models, reinforcement learning, long-form generation, credit assignment

## Abstract

**Highlights:**

**What are the main findings?**

- Proposes AF-CuRL, a lightweight reinforcement learning framework that improves training stability for long-form generation under low-resource constraints.
- Demonstrates consistent gains in mathematical reasoning accuracy and output regularity on a 1.5B-parameter model without increasing model size or compute.

**What are the implications of the main findings?**

- Shows that objective-level design, rather than model scaling, is critical for effective reinforcement learning in low-resource long-form generation.
- Provides a practical and reproducible reinforcement learning approach applicable to resource-constrained reasoning tasks.

**Abstract:**

Resource-constrained intelligent systems increasingly require reliable long-form reasoning capabilities under limited computational and memory budgets, particularly in edge and embedded sensing environments. However, reinforcement learning for long-horizon decision generation remains highly unstable in such low-resource settings due to severe reward sparsity and imbalanced credit assignment, which often lead to non-convergent or excessively verbose generation behavior. In this work, we propose AF-CuRL (Answer-Focused Curriculum Reinforcement Learning), a lightweight reinforcement learning framework designed to stabilize long-form generation without increasing model size or computational cost. AF-CuRL improves optimization learnability through two complementary objective-level designs: (1) answer-focused token reweighting, which concentrates policy updates on reward-critical regions of generated sequences to alleviate credit assignment imbalance, and (2) a two-phase curriculum reward schedule that prioritizes stable termination and output regularity before shifting toward correctness-oriented optimization. We evaluate AF-CuRL on a 1.5B-parameter language model under strictly constrained training settings, using mathematical reasoning tasks as a controlled and reproducible proxy for long-horizon, rule-based decision-making commonly encountered in intelligent sensing and embedded systems. Experimental results demonstrate consistent improvements in both decision accuracy and generation regularity, including higher termination reliability and reduced generation length, compared with standard sequence-level reinforcement learning baselines. These results suggest that, for resource-limited and edge-intelligent systems, structured objective design can be more effective than model scaling for achieving stable and efficient long-form reasoning, providing a practical reinforcement learning solution for intelligent systems operating under real-world constraints.

## 1. Introduction

In recent years, large language models (LLMs) have achieved substantial progress in natural language understanding and generation, enabling their use not only in traditional AI tasks such as code generation and mathematical reasoning, but also as high-level reasoning and decision modules in intelligent systems. In emerging sensor-driven and edge-intelligent applications, LLMs are increasingly explored as a means to interpret structured observations, perform multi-step symbolic or rule-based reasoning, and produce explicit terminal decisions under complex constraints [1,2,3]. As these systems move closer to practical deployment, reinforcement learning (RL) has become an important paradigm for refining LLM behavior beyond supervised fine-tuning, particularly when explicit labels are unavailable or when decision quality depends on long-horizon outcomes [4,5].

Despite their promise, applying reinforcement learning to LLM-based reasoning modules in sensor-driven and edge-intelligent systems remains fundamentally challenging, particularly under low-resource constraints. A central difficulty arises from the credit assignment problem in long-horizon decision generation: rewards are often available only after an entire reasoning or decision sequence has been completed, while the sequence itself may consist of hundreds or thousands of intermediate tokens [6]. Under such settings, token-level contributions to terminal outcomes are highly imbalanced. Most tokens correspond to intermediate reasoning or structural transitions and contribute weakly to the final decision, whereas a small subset of tokens near the end of the sequence largely determines correctness or task success [7]. When standard sequence-level policy gradient methods propagate identical reward signals across all tokens, gradients correlated with terminal outcomes are diluted by a large number of weakly related decisions, leading to high-variance updates and unstable optimization [8]. These issues become especially pronounced when model capacity, sampling budgets, and variance-reduction mechanisms are simultaneously constrained, a setting that naturally arises in resource-limited and edge-intelligent systems [9].

In this work, mathematical reasoning is used as a controlled benchmark rather than a target application, as it exhibits structured, long-horizon decision characteristics similar to those required in LLM-based reasoning modules for intelligent sensing systems. Mathematical reasoning tasks provide a representative testbed for examining these challenges [10]. On the one hand, models are required to generate relatively long intermediate reasoning sequences to perform multi-step logical deductions [11]. On the other hand, evaluation depends critically on the correctness and format of the final answer produced at the end of the sequence. In practice, models often produce concise and well-defined final answers for problems of moderate difficulty. However, as problem difficulty increases, they are prone to generating excessively long outputs, repeatedly revisiting reasoning steps without converging to a final result. Such behaviors not only degrade evaluation metrics, but also highlight the limitations of existing reinforcement learning methods when applied to long-form generation under constrained resources [12].

Existing reinforcement learning approaches for large language models typically adopt sequence-level reward modeling, treating all tokens in a generated sequence as equally important optimization targets [13]. Moreover, many methods emphasize final-answer correctness as the primary reward signal from the very beginning of training. Under low-resource conditions, this design often results in extremely sparse and unstable reward signals, providing little effective supervision during early optimization [14]. As a consequence, policy updates are dominated by noise, leading to unstable and non-convergent generation behavior rather than sustained performance improvement under low-resource conditions [15,16].

Motivated by these observations, we propose AF-CuRL (Answer-Focused Curriculum Reinforcement Learning), a reinforcement learning framework designed specifically for low-resource long-form generation. The central objective of AF-CuRL is to stabilize reinforcement learning by imposing structured constraints on the optimization objective and training signals, while leaving the underlying model architecture unchanged. Rather than relying on increased sampling budgets, auxiliary critic networks, or large-scale computational resources, AF-CuRL addresses the core bottlenecks of long-form reinforcement learning directly at the objective level.

Specifically, AF-CuRL improves reinforcement learning from two complementary perspectives. First, it introduces answer-focused token reweighting, which assigns greater optimization emphasis to answer-critical regions of the generated sequence, thereby alleviating credit assignment imbalance in sequence-level policy gradients. Second, it employs a two-phase curriculum reward schedule that prioritizes stable and learnable behavioral signals—such as output format and generation length—during early training, before gradually shifting emphasis toward correctness-oriented optimization once generation behavior has stabilized [17].

AF-CuRL is implemented using a lightweight REINFORCE-based training paradigm and relies exclusively on parameter-efficient fine-tuning via LoRA adapters and rule-based reward functions [18,19,20]. No additional value networks or complex optimization architectures are introduced. As a result, the proposed framework can be trained on a single consumer-grade GPU with limited memory, making it suitable for practical scenarios involving constrained model capacity and restricted training budgets.

Using mathematical reasoning as the primary evaluation domain, we conduct systematic experiments on a 1.5B-parameter language model under strictly low-resource conditions [21]. Experimental results demonstrate that AF-CuRL achieves consistent and reproducible improvements across multiple mathematical reasoning benchmarks. Further ablation studies and behavioral analyses confirm that the proposed design not only improves final correctness, but also systematically enhances output regularity and generation efficiency in long-form reasoning tasks.

In summary, this work makes the following contributions:

1. We introduce an answer-focused policy optimization formulation that explicitly addresses credit assignment imbalance in long-form generation.
2. We propose a two-phase curriculum reward scheduling strategy that substantially improves training stability under low-resource conditions.
3. We demonstrate, through systematic experiments on a 1.5B-parameter model trained on a single GPU, that structured objective design enables stable and reproducible reinforcement learning improvements without increasing model scale or computational cost.

## 2. Related Work

### 2.1. Reinforcement Learning for Large Language Models

Reinforcement learning has been widely applied to the post-training and alignment of large language models, particularly following supervised fine-tuning (SFT) [22], to further optimize generation behavior and alignment with human preferences. Representative approaches include reinforcement learning from human feedback (RLHF) frameworks based on Proximal Policy Optimization (PPO) [23,24,25,26], as well as subsequent variants that incorporate reference policies and KL regularization to improve training stability [27].

From an optimization perspective, these approaches typically model large language models as autoregressive stochastic policies and perform parameter updates using sequence-level reward signals through policy gradient or actor–critic methods [28,29]. To mitigate the high variance of gradient estimation, prior work commonly relies on large batch sizes, multiple samples per input, and explicit value functions or critic networks [24]. Under settings with substantial computational resources and large model capacity, this paradigm has been shown to be effective across a wide range of tasks [30].

However, these methods implicitly rely on several assumptions that are difficult to satisfy under low-resource conditions. In particular, they presume sufficient sampling budgets, stable and well-trained critic networks, and reward signals that are dense enough to support early-stage learning. When these assumptions break down—such as in scenarios involving constrained model capacity, limited sampling (e.g., single-sample updates), or the absence of auxiliary critic networks—training stability can deteriorate substantially.

Recent work has explored alternatives to actor–critic frameworks, including policy gradient methods without explicit value estimation, such as REINFORCE with baselines or group-based policy optimization (GPRO) [31]. While these approaches reduce architectural complexity and improve reproducibility, they still predominantly operate on sequence-level reward signals and do not explicitly address the credit assignment imbalance inherent in long-form generation. However, how to design reinforcement learning objectives that explicitly address token-level contribution imbalance in low-resource settings has received little direct attention so far.

### 2.2. Structural Limitations of Low-Parameter Language Models

Under constrained parameter settings, large language models often exhibit a range of structural limitations in long-form generation tasks. These limitations are not primarily reflected in insufficient reasoning capability, but rather in the difficulty of achieving stable convergence in generation behavior. Prior studies have shown that small-parameter models face inherent bottlenecks in long-range dependency modeling; even when longer context windows are formally supported, the ability to retain and utilize early generated information degrades significantly as sequence length increases.

In long-horizon logical reasoning tasks, this limitation typically manifests as reasoning drift: as the number of reasoning steps increases, early intermediate conclusions are gradually forgotten or overwritten, and local generation errors accumulate, ultimately making it difficult for the model to determine when to terminate reasoning and produce a final answer [32]. Empirically, such models tend to “delay decision-making” by generating excessively long text, thereby avoiding the production of explicit and well-defined final results. This form of non-terminating or overly verbose generation is particularly common in mathematical reasoning tasks. These behavioral tendencies motivate the need for reinforcement learning objectives that explicitly regularize termination and output structure, as discussed in the following sections [33].

### 2.3. Credit Assignment in Long-Form Generation

Credit assignment is a classical challenge in reinforcement learning, particularly in settings where rewards are provided only after long temporal delays [34]. In long-form generation tasks, this problem exhibits a more structured form: generated sequences typically contain a large number of intermediate tokens, while the reward is often determined by only a small number of decisions near the end of the sequence [35].

In the context of long-form generation with large language models, the difficulty of credit assignment does not stem solely from sequence length, but from the extreme imbalance in token-level contributions to the terminal reward. In mathematical reasoning tasks, the vast majority of tokens are devoted to intermediate reasoning and contribute negligibly to final correctness, whereas a small number of tokens associated with the final answer almost entirely determine the reward value. This highly uneven contribution structure causes conventional sequence-level policy gradients to implicitly assign equal credit to all tokens, introducing substantial misalignment at the optimization level.

Specifically, when policy gradient updates are applied uniformly across the entire generated sequence, gradients that are highly correlated with the reward are diluted by a large number of weakly related tokens, causing the variance of gradient estimation to grow substantially with sequence length. This effect is particularly pronounced under low-resource conditions: when sampling is limited (e.g., K = 1) and critic networks or auxiliary value estimators are unavailable, the statistical efficiency of policy gradients depends critically on the correlation structure between reward signals and generation decisions [24].

Although prior studies have recognized the difficulty of credit assignment in long-form generation, this issue is often partially obscured in large-model or high-compute settings through the use of multi-sample strategies, large batch sizes, or actor–critic architectures. In low-resource regimes, however, these mitigation mechanisms are difficult to maintain, and token-level credit imbalance translates directly into amplified gradient noise, substantially weakening training stability and learnability. As a result, explicitly modeling credit assignment without introducing additional model structures remains one of the central challenges in low-resource long-form reinforcement learning.

### 2.4. Curriculum Learning and Reward Scheduling

Curriculum learning guides model convergence by progressively adjusting the difficulty of the training process and has been extensively studied in both supervised and reinforcement learning settings [36,37]. In reinforcement learning, prior work typically organizes training into stages based on task difficulty or environmental complexity, or employs reward shaping to provide auxiliary signals for intermediate behaviors, with the aim of improving sample efficiency and training stability [38].

However, most existing curriculum learning approaches are organized around task difficulty or environmental complexity, implicitly assuming that the reward signal remains sufficiently learnable at each training stage. In low-resource long-form generation settings, this assumption often does not hold. In particular, when rewards are primarily determined by terminal correctness, small-parameter models receive little effective positive feedback during early training, rendering policy gradient updates that directly optimize for correctness highly unstable.

In contrast to curriculum learning strategies driven by task difficulty, this work focuses on curriculum reward scheduling at the level of the reward objective. The central idea is not to modify the task or data distribution, but to improve the learnability of policy gradients by optimizing different reward components at different stages of training. During early training, greater emphasis is placed on objectives related to convergence of generation behavior, such as output format and generation length, in order to increase reward density and stabilize policy updates; once stable termination behavior has been established, optimization gradually shifts toward correctness-oriented objectives.

This design is fundamentally different from conventional reward shaping. Traditional reward shaping typically smooths or reweights rewards within a single training stage, whereas curriculum reward scheduling addresses stage-specific training bottlenecks by progressively shifting the optimization focus across training phases. Under low-resource conditions, such stage-wise objective transitions substantially mitigate reward sparsity and gradient noise, providing a necessary foundation for subsequent correctness improvement. As a result, curriculum reward scheduling offers an effective alternative to task-based curricula for low-resource long-form reinforcement learning.

## 3. Method

**Figure 1:**
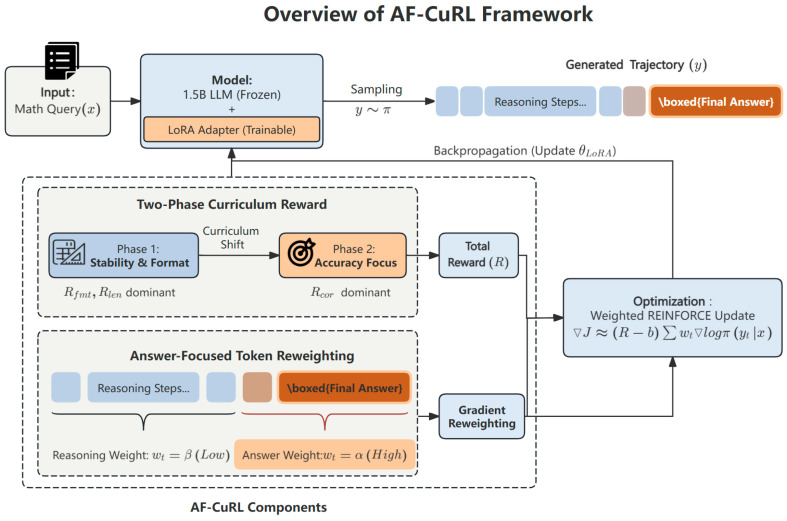
AF-CuRL optimizes a frozen LLM with LoRA adapters using an answer-focused REINFORCE objective. The framework combines token-level reweighting with a two-phase curriculum reward to improve credit assignment and training stability in long-form generation.

This section introduces AF-CuRL (Answer-Focused Curriculum REINFORCE), the reinforcement learning method proposed in this work. AF-CuRL is designed for reinforcement learning training of large language models under low-resource conditions, with the goal of guiding models toward more regularized, convergent, and efficient generation behavior without relying on complex optimizers or substantial computational resources, as illustrated in Figure 1. The framework consists of three components:

1. a lightweight training paradigm based on REINFORCE;
2. an answer-focused token reweighting strategy;
3. a two-phase curriculum reward mechanism.

### 3.1. Task Formulation and Reinforcement Learning Setup

We consider the problem of training large language models for long-form generation using reinforcement learning under low-resource conditions. Given an input prompt x, the model generates an output sequence $y=(y_{1},\ldots ,y_{T})$ in an autoregressive manner, where T denotes the length of the generated sequence [39]. The model is treated as a stochastic policy πθ parameterized by θ, whose conditional probability distribution is defined as:

(1) $$\pi_{\theta }\;(y|x)=\prod_{t=1}^{T}\pi_{\theta }\;(y_{t}|y_{<t},x)$$

Under the reinforcement learning framework, the training objective of the model is to maximize the expected reward of the generated sequence:

(2) $$\underset{\theta }{\max}\;E_{y∼\pi_{\theta }(\cdot |x)}\;[R(x,y)]$$

In the standard REINFORCE algorithm, this objective corresponds to the following policy gradient loss function:

(3) $$L_{REINFORCE}\;(\theta )=-R(x,y)\sum_{t=1}^{T}\log\pi_{\theta }\;(y_{t}|y_{<t},x)$$

Here, the reward function R(x,y) is computed after the complete sequence has been generated and is used to evaluate the overall quality of the model output. In the setting studied in this work, reward signals are not provided at the token level, but are instead defined at the sequence level.

Under the low-resource setting, we adopt a K=1 sampling strategy for reinforcement learning training, where a single output sequence is generated for each input at every training step and the policy gradient update is computed based solely on that sequence. To maintain simplicity and reproducibility, we do not introduce additional value functions or critic networks, and all parameter updates are applied directly to the language model policy itself.

It is important to note that we distinguish between sampling strategies used during training and evaluation. Reinforcement learning training is consistently performed with K=1 sampling, whereas during evaluation we allow multiple samples per input and aggregate results using a best-of-K strategy [40]. This evaluation protocol is used solely to reduce stochasticity at inference time and does not affect the training objective or the proposed method itself.

### 3.2. Limitations of Sequence-Level Policy Gradients in Long-Form Generation

Building on the analysis of credit assignment imbalance discussed in Section 2.3, we briefly restate the limitation of sequence-level policy gradients most relevant to our method. Although sequence-level policy gradients such as REINFORCE provide an unbiased estimator of the expected reward objective, their optimization behavior exhibits fundamental limitations in long-form generation settings [39]. Under the standard REINFORCE formulation, the policy gradient estimator can be written as:

(4) $$\nabla_{\theta }L(\theta )=-(R-b)\;\nabla_{\theta }\log\pi_{\theta }\;(y|x)$$

where R denotes the sequence-level terminal reward and b is a baseline used for variance reduction. This estimator assigns the same reward signal to all tokens in the generated sequence through the joint log-probability logπθ(y∣x), implicitly assuming that each token contributes equally to the terminal reward.

When the generated sequence is long, the vast majority of tokens serve intermediate reasoning or linguistic transition roles and contribute negligibly to the final outcome, while only a small subset of tokens near the end of the sequence, typically associated with the final answer, largely determines the terminal reward. Under this highly imbalanced token-level contribution structure, uniformly propagating the reward signal across the entire sequence causes gradients that are strongly correlated with the terminal reward to be diluted by a large number of weakly related tokens. As a result, the effective signal-to-noise ratio of gradient estimates degrades rapidly as sequence length increases.

These limitations become particularly severe under low-resource conditions. When sampling is restricted (e.g., K = 1), model capacity is limited, and no critic or auxiliary value estimation mechanisms are available, policy updates rely almost entirely on a single generated trajectory. In this regime, the statistical efficiency of policy gradients is dominated by the correlation structure between reward signals and generation decisions. Applying uniform sequence-level updates further amplifies gradient noise, making it difficult for the model to obtain stable and informative learning signals during early training.

Empirically, this instability often manifests as non-convergent or delayed generation behavior. These behaviors are not incidental but arise as a structural consequence of applying standard sequence-level policy gradients to long-form generation under low-resource constraints. Therefore, relying solely on conventional REINFORCE updates is insufficient for stable training in this setting, motivating the need for objective designs that explicitly account for token-level contribution imbalance in long-form generation.

### 3.3. Answer-Focused Token Reweighting

#### 3.3.1. Motivation

As discussed in Section 3.2, the central limitation of sequence-level policy gradients in long-form generation lies in the highly imbalanced token-level contributions to the terminal reward. Standard REINFORCE applies a uniform weight to all tokens at the objective level, implicitly assuming that each token is equally important to the final outcome. This assumption substantially amplifies gradient noise and undermines training stability.

**Figure 2:**
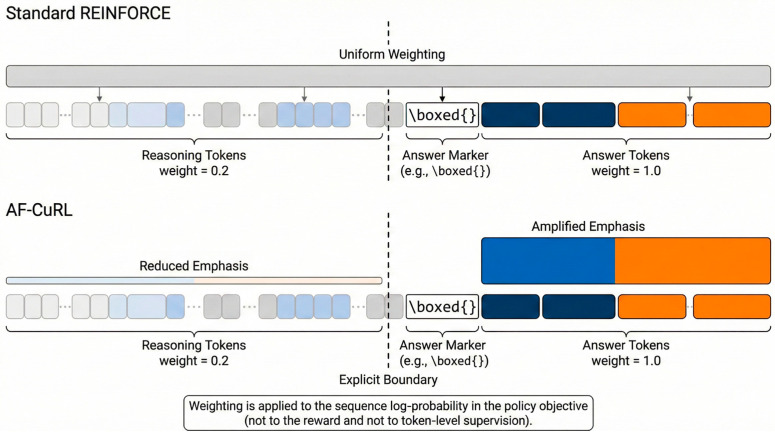
Answer-focused token reweighting in AF-CuRL.

Based on this observation, we propose an answer-focused token reweighting strategy whose goal is not to alter the form of the gradient estimator itself, but to structurally reparameterize the policy optimization objective so that policy updates are concentrated on generation regions that are decisive for the terminal reward, as illustrated in Figure 2.

In tasks such as mathematical reasoning, model outputs can typically be divided into two categories of tokens: (1) reasoning tokens used to construct intermediate reasoning steps or organize language, and (2) answer tokens that express the final result. The latter usually appear near the end of the sequence and are identified by explicit syntactic boundaries, such as the first occurrence of \boxed{…}. Importantly, this partition relies solely on positional and syntactic cues in the output structure, and does not use the numerical value, semantic content, or correctness of the answer, thereby avoiding any task-specific oracle.

#### 3.3.2. Answer-Focused Token Weighting

As discussed in Section 3.2, the main limitation of sequence-level policy gradients in long-form generation lies in the highly imbalanced contribution of individual tokens to the terminal reward. In tasks such as mathematical reasoning, only a small subset of tokens near the end of the sequence—corresponding to the final answer—largely determines the reward, while most preceding tokens contribute weakly.

To address this issue, we introduce an answer-focused token weighting scheme that modulates the contribution of different tokens directly within the policy optimization objective. Specifically, given a generated sequence y = (y_1_, …, y_T_), we identify an answer region starting at position ta, which corresponds to the first occurrence of an explicit answer delimiter (e.g., \boxed{}) [41].

We assign a scalar weight $w_{t}$ to each token position t as follows:

(5) $$w_{t}=\left\{\begin{array}{ll} \alpha , & t\geq t_{a}\\ \beta , & t<t_{a} \end{array}\right.$$

In our experiments, the weighting parameters are fixed as follows:

(6) α=1.0, β=0.2

In our experiments, the weighting coefficients are fixed as α = 1.0 and β = 0.2 across all datasets and training runs. These values are not obtained through task-specific hyperparameter tuning but are chosen as a simple and stable heuristic to reflect the highly imbalanced contribution of different token regions to the terminal reward in long-form generation.

The absolute numerical values of α and β are not critical; rather, the relative scale between answer and non-answer tokens is the key factor. As long as answer-region tokens receive substantially higher weight than reasoning tokens, the optimization behavior remains consistent. We deliberately adopt fixed and interpretable coefficients to avoid introducing additional tuning freedom and to ensure reproducibility under low-resource training conditions.

#### 3.3.3. Weighted REINFORCE Loss Function

Incorporating the answer-focused token weights into the policy gradient formulation, we define the weighted REINFORCE loss used in AF-CuRL as:

(7) $$L_{AF}\;(\theta )=-R(x,y)\sum_{t=1}^{T}w_{t}\log\pi_{\theta }\;(y_{t}|y_{<t},x)$$

where R denotes the sequence-level terminal reward, b is a baseline for variance reduction, and πθ is the autoregressive policy defined in Section 3.1.

Compared to the standard sequence-level REINFORCE objective, this formulation explicitly concentrates policy updates on answer-critical regions of the generated sequence, while down-weighting tokens that are weakly correlated with the terminal reward. Although this modification introduces bias relative to the original unbiased policy gradient estimator, it substantially improves the signal-to-noise ratio of gradient updates in low-resource long-form generation settings.

In practice, we find that this objective leads to more stable training dynamics and more reliable termination behavior, without introducing additional model components or increasing computational overhead.

### 3.4. Two-Phase Curriculum Reward Mechanism

#### 3.4.1. Reward Sparsity Under Low-Resource Conditions

In low-resource long-form generation tasks, another central bottleneck in reinforcement learning training arises from the severe sparsity of reward signals. When rewards are primarily determined by terminal correctness, small-parameter models often receive little to no positive feedback during early training, causing policy gradient updates to be dominated by noise. This issue is particularly pronounced in tasks such as mathematical reasoning: even when a model generates extensive reasoning text, it may still receive zero reward if a clear final answer is not produced.

Taken together with the analyses in Section 3.2 and Section 3.3, it becomes clear that, in long-form generation settings, reward sparsity and imbalanced credit assignment interact and reinforce each other, rendering training that optimizes solely for correctness highly unstable under low-resource conditions. Consequently, providing the policy with denser and more learnable feedback during early training is a necessary prerequisite for achieving stable optimization.

#### 3.4.2. Two-Phase Curriculum Reward Design

**Figure 3:**
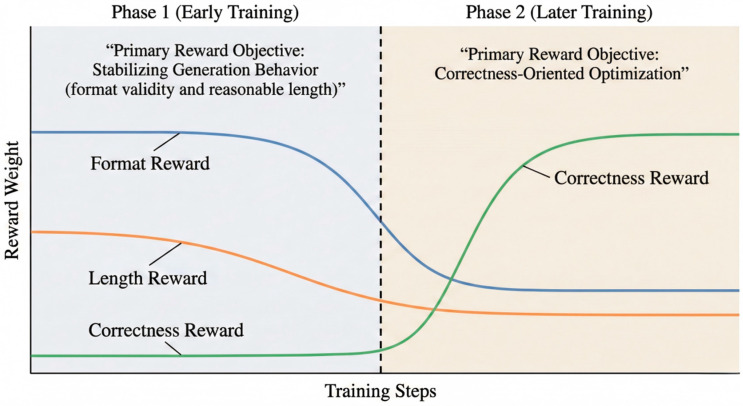
Two-phase curriculum reward schedule in AF-CuRL. AF-CuRL adopts a stage-wise curriculum at the reward-objective level to improve training stability under low-resource conditions. Phase 1 prioritizes dense behavioral rewards (format validity and reasonable length), while Phase 2 shifts emphasis toward correctness-oriented optimization, retaining non-zero structural constraints.

Based on the above observations, we propose a two-phase curriculum reward scheduling mechanism that improves policy gradient learnability by shifting the optimization focus across training stages (Figure 3).

Phase 1: During the early stage of training, the reward function primarily focuses on the determinacy and convergence of generation behavior, rather than optimizing for final correctness. Specifically, this phase emphasizes whether the model produces a final answer with a valid format, and whether the generated sequence length falls within a reasonable range so as to avoid unbounded reasoning expansion. The objective of this phase is not to directly improve task accuracy, but to guide the model toward stable termination behavior and increase reward density, enabling policy gradients to receive reliable positive learning signals.

Phase 2: Once the model has developed stable output patterns, the reward function gradually shifts toward emphasizing correctness, while retaining constraints on output format and generation length. At this stage, the policy has acquired the ability to produce well-defined answers, and correctness-based rewards are no longer highly sparse, allowing policy gradients to more effectively leverage outcome feedback to drive performance improvement.

#### 3.4.3. Reward Function Formulation

Under the curriculum design described above, the reward function used in this work can be expressed as:

(8) $$R(x,y)=\alpha \cdot R_{correct}(x,y)+\beta \cdot R_{format}(y)+\gamma \cdot R_{length}(y)$$

where the optimization focus of the reward function is adjusted across training phases by varying the coefficients α, β, γ. Without introducing additional models or parameters, this curriculum reward mechanism significantly improves the stability of reinforcement learning training under low-resource conditions by enabling a stage-wise transition of optimization objectives.

This curriculum mechanism is not a simple reweighting or smoothing of rewards; rather, it addresses the dominant bottlenecks at different stages of training by shifting the primary optimization focus over time. Under low-resource conditions, such optimization target scheduling effectively mitigates reward sparsity and gradient noise during early training, providing a stable foundation for subsequent correctness-oriented optimization [41].

## 4. Implementation Details

### 4.1. Model and Computational Resources

All reinforcement learning experiments in this work are conducted on an open-source large language model with approximately 1.5B parameters (DeepSeek-R1-Distill-Qwen-1.5B) [42]. This model serves as a representative low-parameter LLM and is used to study the stability and learnability of reinforcement learning training under constrained model capacity and computational resources. This model scale is also chosen with future deployment in resource-constrained or edge-computing environments in mind, where model size, memory footprint, and training stability are critical practical considerations.

To ensure fairness and reproducibility, all comparative methods in this study are implemented using the same base model, the same tokenizer, and an identical inference interface. Training configurations are strictly constrained to settings that can be executed on a single consumer-grade GPU with 8 GB of memory, thereby avoiding implicit advantages from high-compute resources and ensuring that the experimental conditions closely reflect practical and reproducible low-resource scenarios.

During training, we adopt a parameter-efficient fine-tuning (PEFT) strategy, updating the model exclusively through LoRA adapters while keeping the base model parameters frozen [43]. This design substantially reduces memory and computational overhead, and also helps mitigate instability arising from large-scale parameter updates in low-resource reinforcement learning.

### 4.2. Datasets and Task Setup

We adopt mathematical reasoning as the evaluation setting because it simultaneously exhibits three key characteristics: (1) the requirement for long-form generation, (2) strict constraints on final answers, including numerical correctness and output format, and (3) common forms of generation behavior degradation in high-difficulty cases, such as excessively long reasoning, repeated attempts, or failure to produce a final answer. These properties make mathematical reasoning a representative testbed for assessing the stability of reinforcement learning training under low-resource conditions.

We strictly distinguish the use of data between the reinforcement learning training phase and the evaluation phase:

- RL-train: Used for reinforcement learning training and contains only a small number of samples, reflecting the low-resource setting.
- Dev: Used for monitoring training trends and model selection, without participating in gradient updates.
- Test/Benchmarks: Used for reporting final results. In addition to the primary test set, we conduct zero-shot evaluations on multiple external mathematical benchmarks to assess generalization performance.

All data splits follow a strict separation between training, development, and testing to prevent any leakage of training data into the evaluation stage.

### 4.3. Reinforcement Learning Training Setup

#### 4.3.1. Algorithm and Sampling Configuration

We adopt REINFORCE (policy gradient) as the base training paradigm and perform training under low-resource constraints using a K = 1 sampling strategy. At each training step, a single output sequence is generated for each sample, and policy gradient updates are computed based on the corresponding sequence-level reward. No additional value networks or critic structures are introduced, avoiding the instability and implementation complexity associated with auxiliary modules and highlighting the effectiveness of AF-CuRL under a simple and reproducible training setup. All comparison methods in this study, including Plain RL and ablation variants, employ the same sampling strategy and optimization paradigm, namely K = 1 sampling, no critic network, and identical policy gradient updates, thereby ensuring fair comparisons without confounding effects from differing training protocols.

#### 4.3.2. Training Steps and Curriculum Phases

Training follows a two-phase curriculum reward schedule (Phase 1/Phase 2), with phase transitions determined by the number of training steps. Phase 1 emphasizes the learnability of generation behavior, such as constraints on output format and generation length, while Phase 2 progressively increases the emphasis on correctness once generation behavior has stabilized. This curriculum design aims to mitigate early-stage training instability caused by reward sparsity under low-resource conditions. The division between curriculum phases is based solely on training steps rather than performance metrics, and is kept fixed across all datasets and experimental configurations, without task-specific tuning of phase boundaries.

#### 4.3.3. Stability Techniques and Reproducibility Constraints

Because low-resource reinforcement learning is highly sensitive to randomness and gradient noise, we apply several standard stabilization techniques to improve training controllability and reproducibility. Specifically, all experiments use fixed random seeds, gradient clipping, and an exponential moving average (EMA) reward baseline to reduce the variance of policy gradient estimates.

To ensure fair comparisons, these stabilization techniques are applied consistently across all methods and ablation settings, without introducing any additional stabilization specific to AF-CuRL. During training, we periodically evaluate the model on a validation set to monitor potential issues such as generation behavior degradation or training collapse, although validation results are not used for gradient updates.

The transition between Phase 1 and Phase 2 is determined solely by the number of training steps and is kept fixed across all datasets and experimental settings. This design choice is intentional and aims to maximize reproducibility under low-resource reinforcement learning conditions. Although performance-based criteria such as boxed rate stabilization could in principle be used to trigger phase transitions, such metrics are highly noisy during early-stage training and often require task-dependent thresholds. Introducing performance-based switching would therefore add additional heuristic complexity and tuning freedom. In contrast, a fixed step-based schedule provides a simple, stable, and easily reproducible curriculum structure without relying on task-specific performance signals.

### 4.4. Parameter-Efficient Fine-Tuning Configuration

We employ LoRA (Low-Rank Adaptation) for parameter-efficient fine-tuning, updating only a small number of low-rank adapter parameters while keeping the base model weights frozen. This design offers three key advantages: (1) it substantially reduces memory and computational overhead, enabling training on a single 8GB GPU; (2) it limits the number of trainable parameters, which contributes to training stability under low-resource conditions; and (3) it improves reproducibility, facilitating fair comparisons under consistent experimental settings.

The LoRA rank, scaling factor, and dropout are fixed across all experiments to ensure implementation symmetry among different methods. Aside from LoRA, we do not modify the base model architecture or introduce additional supervision heads or discriminator modules.

### 4.5. Reward Function and Evaluation Signal

We adopt a rule-based, sequence-level reward function whose components and formulation strictly follow the definition in Section 3.4. The reward function consists of interpretable components, including correctness, output format, and length or efficiency, and is designed to jointly regulate output quality and generation behavior.

To ensure reproducibility and interpretability, no learned reward models or preference models are used. All reward signals are computed using deterministic rules and evaluation scripts, and the coefficients associated with each reward component remain fixed throughout training, without dataset-specific or configuration-specific tuning.

The implementation of the reward function serves solely to realize the curriculum reward scheduling mechanism described in the Section 3, rather than to introduce additional engineering heuristics or implicit optimization objectives. Detailed training configurations and implementation settings are provided in Appendix A.

### 4.6. Evaluation Protocol and Metrics

#### 4.6.1. Distinction Between Training and Evaluation Sampling

To remain consistent with the low-resource training setup, reinforcement learning training is conducted using fixed K = 1 sampling. During evaluation, we allow multiple samples per input and aggregate results using a best-of-K strategy, which reduces variance caused by stochastic generation and yields more stable and comparable results. Multiple sampling is used exclusively at evaluation time and does not alter the training objective or the training procedure [12].

#### 4.6.2. Metrics

The primary metric reported in this work is pass@K (also referred to as best-of-K accuracy), which measures the model’s ability to produce a correct answer across multiple sampled generations. In addition, we report behavior-related metrics to characterize the impact of reinforcement learning on generation patterns, including but not limited to:

- Boxed rate: the proportion of outputs that contain a properly formatted final answer.
- Generation length: the length of the generated output, used to assess generation efficiency and convergence.
- Additional length statistics related to reasoning segments, when explicit reasoning markers are present in the output.

Together, these metrics support the central claim of this work that AF-CuRL not only improves outcome-level performance, but also enhances the regularity and efficiency of generation behavior.

#### 4.6.3. Prompt Consistency

To ensure fair comparisons, all methods—including baselines, plain RL, and AF-CuRL—use the same prompt construction logic and identical decoding parameters. Input prompts are generated uniformly within the evaluation scripts and used consistently for sampling across methods, thereby eliminating confounding factors arising from prompt variations. The prompt used in all experiments is as follows:

“You are an expert competition mathematician.”
“Directly give the final numerical answer in the format \\boxed{}.”
“Do NOT show any reasoning steps.”

The prompt is intentionally kept simple and is designed solely to standardize input and output formats, without incorporating explicit reasoning strategies or task-specific guidance. The same prompt is used during both training and evaluation for all methods, preventing prompt engineering from influencing comparative results.

Although the prompt instructs the model not to explicitly output reasoning steps, models may still generate lengthy intermediate text on high-difficulty problems. The analysis of long-form generation behavior in this work is based on such naturally emerging outputs, rather than on explicit chain-of-thought prompting.

### 4.7. Implementation and Reproducibility

All experiments in this work are implemented within a unified code framework (e.g., the PyTorch ecosystem), with key sources of randomness fixed, including random seeds, data sampling procedures, and evaluation protocols. Given the sensitivity of reinforcement learning training to hyperparameters, we adopt a reproducibility-first design principle that deliberately limits tuning flexibility. Training-related hyperparameters, such as learning rate, batch size, and the number of training steps, are primarily determined by low-resource hardware constraints and commonly used ranges for parameter-efficient fine-tuning [43]. Method-specific hyperparameters, such as the relative weighting between answer and non-answer regions, emphasize relative scale relationships rather than finely tuned optimal values, thereby avoiding unfair gains from repeated experimentation on test data.

In addition, implementation symmetry is strictly maintained across all comparative experiments, including the use of the same base model, tokenizer, prompt, evaluation and reward logic, and evaluation protocol. This ensures that observed correctness improvements can be attributed to the design of AF-CuRL itself, rather than to auxiliary engineering techniques or implementation differences.

## 5. Results

### 5.1. Main Results

Table 1 summarizes the main experimental results of AF-CuRL across multiple mathematical reasoning benchmarks. All methods are evaluated using the same 1.5B-parameter base model and an identical evaluation protocol to ensure fair and reproducible comparisons. Rather than focusing on large-scale reinforcement learning settings, our study examines whether reinforcement learning can stably improve generation behavior and task performance under low-resource constraints, where sampling budgets, model capacity, and optimization structures are all limited.

Under this setting, AF-CuRL consistently outperforms baseline methods across all evaluated benchmarks. Compared with the base model without reinforcement learning, AF-CuRL achieves clear improvements on standard mathematical reasoning datasets such as GSM8K and MATH-500. These results indicate that even under constrained model capacity, structured objective design enables reinforcement learning to yield stable and reproducible performance gains.

Notably, the observed correctness improvements are not confined to a single dataset type. AF-CuRL exhibits consistent gains on benchmarks with diverse problem styles and difficulty distributions, including Minerva, Olympiad-Bench, and AMC23. Although these benchmarks differ substantially in problem structure, reasoning depth, and answer formats, they share a common characteristic: models must generate extended reasoning sequences and produce a strictly verifiable final answer at the end of the sequence.

The consistent performance of AF-CuRL across these benchmarks suggests that its advantage does not stem from specialization to particular problem types or data distributions. Instead, it arises from addressing fundamental bottlenecks in reinforcement learning for long-form generation, particularly those related to credit assignment and reward sparsity.

Table 1 also includes a comparison with an external model of similar parameter scale, OpenMath-Nemotron-1.5B [44]. In this comparison, AF-CuRL achieves competitive results across all reported benchmarks, further supporting the conclusion that its correctness improvements do not rely on increased model scale, but on structured reinforcement learning objectives.

It should be noted that the results in Table 1 reflect only final task performance. The impact of AF-CuRL on generation behavior, such as output regularity and generation efficiency, is examined in subsequent sections through ablation studies and behavioral analyses. While the results demonstrate consistent gains in final correctness, correctness alone is insufficient to fully explain the advantages of AF-CuRL in low-parameter regimes. In high-difficulty mathematical reasoning tasks, multiple methods may exhibit similarly low accuracy, making outcome-based metrics inadequate for capturing differences in generation strategies. Given that AF-CuRL explicitly introduces structured constraints on generation behavior during training, the following section analyzes model outputs from a behavioral perspective, focusing on output regularity and generation efficiency to provide a more comprehensive understanding of how AF-CuRL shapes generation patterns across different difficulty levels.

### 5.2. Ablation Studies

This section presents a systematic ablation study to analyze the contributions of the key components in AF-CuRL to both correctness improvement and the stability of generation behavior. The goal of these experiments is not to identify an optimal configuration, but to verify whether the two core designs—answer-focused token reweighting and the two-phase curriculum reward mechanism—provide complementary and indispensable benefits for reinforcement learning training under low-resource conditions.

All ablation experiments are conducted under settings identical to the main experiments, including the same base model, training data, number of training steps, and evaluation protocol. No additional hyperparameter tuning is performed for any individual configuration, ensuring that performance differences reflect only structural variations in the training objective.

Table 2 summarizes the results of different configurations on the GSM8K and MATH-500 benchmarks. “Plain RL” corresponds to standard sequence-level REINFORCE without answer-focused weighting or curriculum reward scheduling and serves as an unstructured baseline. “Answer-only” introduces token reweighting for the answer region without curriculum rewards. “Curriculum-only” applies the two-phase reward schedule without answer-focused weighting. “AF-CuRL” combines both designs.

The ablation results show that introducing answer-focused token reweighting alone (“Answer-only”) yields consistent correctness improvements on both benchmarks and substantially increases the proportion of outputs that contain properly formatted final answers. This observation indicates that explicitly emphasizing answer-related regions effectively alleviates credit assignment imbalance in sequence-level policy gradients for long-form generation.

Without altering the composition of the reward function, the answer-focused strategy reduces gradient contributions from tokens that are weakly correlated with the terminal reward, thereby concentrating policy updates on regions that decisively affect the final outcome. This reweighting improves the effective signal-to-noise ratio of policy gradients under low-resource conditions.

Applying only the two-phase curriculum reward mechanism (“Curriculum-only”) also outperforms Plain RL across multiple metrics, with particularly pronounced improvements in generation behavior. Compared with configurations without curriculum scheduling, this approach significantly reduces average generation length and increases the frequency with which models produce explicit final answers. These results confirm the role of curriculum reward scheduling in mitigating reward sparsity under low-resource settings. By prioritizing the learnability and convergence of generation behavior during early training, the model receives stable positive feedback earlier, providing a foundation for subsequent correctness-oriented optimization.

When both answer-focused reweighting and curriculum reward scheduling are enabled, the model achieves the best performance across both correctness and behavior-related metrics. This outcome demonstrates that the two designs play complementary roles in reinforcement learning training: answer-focused reweighting improves the structure of credit assignment, while curriculum reward scheduling enhances overall training stability. Their combination more effectively guides models toward regularized and efficient generation behavior under low-resource constraints, enabling stable and reproducible correctness improvements without increasing model size or training budget.

### 5.3. Behavior Analysis

As discussed in Section 5.1, in high-difficulty mathematical reasoning tasks, relying solely on final correctness is often insufficient to fully characterize the differences between training methods. This limitation is particularly evident under low-parameter and low-resource reinforcement learning settings, where the accuracy of most methods may simultaneously approach zero. In such cases, outcome-based metrics alone fail to capture substantive changes in generation strategies.

Since AF-CuRL explicitly introduces structured constraints on generation behavior during training, this section presents a systematic behavioral analysis of model outputs. We focus on whether models are able to produce well-defined and verifiable final answers within a reasonable sequence length. The primary behavioral metrics considered are boxed rate, which measures the presence of an explicit final answer, and generation length, which reflects generation efficiency and convergence. Together, these metrics provide a finer-grained view of behavioral differences across methods in long-form generation settings.

#### 5.3.1. Output Regularity Analysis (Boxed Rate)

We first analyze the proportion of outputs that contain a properly formatted final answer across different benchmarks, as measured by the boxed rate. Specifically, this metric evaluates whether the model produces a valid final answer in the form of \boxed{..}upon completing its reasoning process. The boxed rate reflects whether a model has developed the habit of concluding a solution with an explicit and verifiable answer, rather than remaining in prolonged or unconverged intermediate reasoning.

AF-CuRL consistently improves output completion behavior while producing more concise responses, especially on high-difficulty benchmarks where accuracy alone is insufficient to distinguish model performance.

**Figure 4:**
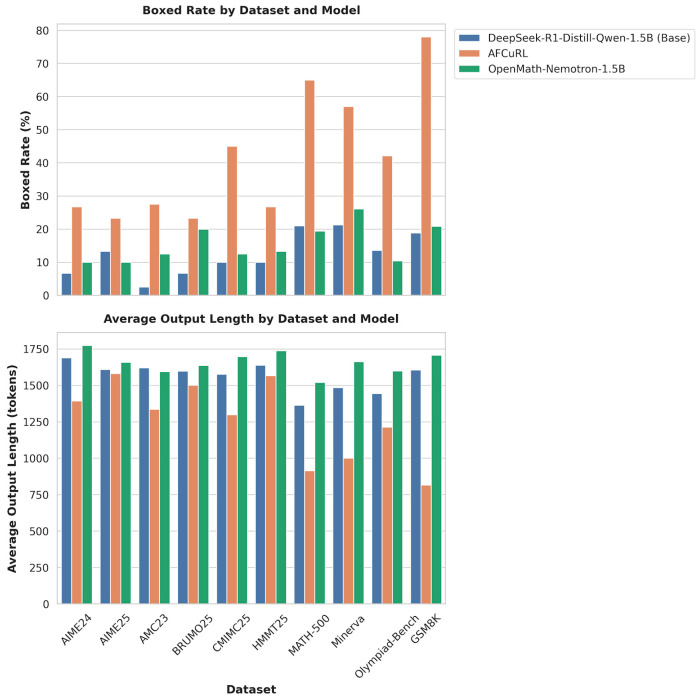
Behavior analysis across multiple math benchmarks. (**Top**) Boxed rate, measuring the proportion of outputs that contain a valid final boxed answer. (**Bottom**) Average output length of generated solutions.

As shown in Figure 4(top), we report the boxed rate of different methods across benchmarks, measuring whether models produce an explicit and properly formatted final answer during generation. Compared with Plain RL and the model trained with supervised fine-tuning only, AF-CuRL consistently achieves substantially higher boxed rates on all benchmarks. This indicates that AF-CuRL-trained models are more likely to conclude reasoning with a verifiable final answer, rather than continuing to generate intermediate reasoning text without convergence. This behavioral difference is closely tied to the design of the AF-CuRL training objective. By explicitly reweighting answer-region tokens in the policy gradient objective, optimization is more directly guided toward the formation of terminal outputs. In addition, the curriculum reward mechanism prioritizes termination behavior and output regularity during early training, enabling the model to learn when to stop reasoning and present a final answer. The increase in boxed rate therefore suggests that AF-CuRL encourages models to terminate generation more reliably under low-resource training, instead of continuing unbounded reasoning.

More notably, on extremely challenging competition-level benchmarks such as AIME, AMC, CMIMC, and HMMT, where the final accuracy of all models remains close to zero, AF-CuRL still exhibits a clear advantage in boxed rate. In contrast, the base model more frequently generates long but non-terminating reasoning sequences, whereas AF-CuRL produces well-structured final answers more often. These results suggest that even when model capacity is insufficient to solve the problem correctly, AF-CuRL effectively guides the model toward an output pattern that completes reasoning and delivers an explicit conclusion.

#### 5.3.2. Generation Efficiency Analysis (Generation Length)

Beyond output regularity, we further analyze the average generation length produced by different methods to assess generation efficiency during reasoning. As shown in Figure 4(bottom), AF-CuRL substantially reduces the average generation length across multiple benchmarks, without any accompanying degradation in task performance.

Importantly, shorter generation length does not indicate weakened reasoning capability, but rather reflects earlier convergence in the reasoning process. Compared with other methods, AF-CuRL-generated sequences exhibit fewer instances of redundant repetition or unproductive continuation, thereby avoiding the non-terminating behavior commonly observed in low-resource reinforcement learning. This observation is consistent with the analysis in Section 2.2, which highlights the tendency of low-parameter models to suffer from reasoning drift during long-horizon logical generation.

#### 5.3.3. Behavioral Differences on High-Difficulty Benchmarks

On extremely challenging benchmarks such as AIME, CMIMC, and HMMT, the final accuracy of a 1.5B-parameter model is often too low to serve as a reliable discriminator between different methods. In these settings, behavioral differences become particularly informative.

Considering both boxed rate and generation length, AF-CuRL exhibits more stable and consistent generation behavior on these benchmarks. Models trained with AF-CuRL more frequently produce structurally complete outputs and avoid unbounded reasoning expansion. In contrast, the base model is more prone to divergent reasoning processes, overly verbose outputs, and the absence of clear termination signals.

#### 5.3.4. Summary

Taken together, the behavioral analyses indicate that the advantages of AF-CuRL under low-resource reinforcement learning settings lie primarily in its systematic guidance of generation behavior, rather than in directly expanding the model’s reasoning capability. By improving termination behavior and generation efficiency, the model is able to form well-defined and verifiable output patterns more reliably under limited training budgets, thereby providing a necessary foundation for subsequent correctness-oriented optimization.

These findings suggest that, in low-parameter and long-form generation scenarios, the key challenge in reinforcement learning training is not merely to maximize terminal rewards, but to enable models to first learn how to complete a reasoning process in a controlled and well-structured manner through structured objective design. This observation provides an important basis for the following discussion on the applicability and limitations of the proposed method.

## 6. Discussion and Limitations

### 6.1. Behavioral Stability and Correctness Improvement in Low-Parameter Models

The experimental results demonstrate that AF-CuRL consistently improves final correctness on multiple mathematical reasoning benchmarks for low-parameter language models, while simultaneously enhancing generation behavior. Importantly, these improvements should not be interpreted as an expansion of the model’s inherent reasoning capacity. Instead, the primary contribution of AF-CuRL lies in improving behavioral stability in long-form generation through structured reinforcement learning objective design.

Under low-resource reinforcement learning settings, small-parameter models are often capable of producing meaningful intermediate reasoning steps, but struggle to converge toward well-defined and properly terminated outputs. This instability leads to sparse and noisy training signals, which severely limits the effectiveness of reinforcement learning. By combining answer-focused token reweighting with curriculum reward scheduling, AF-CuRL explicitly guides models to learn reliable termination behavior and to produce properly formatted final answers earlier in training. This behavioral stabilization serves as a prerequisite for effective outcome-level optimization and constitutes the main mechanism through which correctness improvements are achieved.

This effect is particularly evident in low-parameter and high-difficulty regimes, where model capacity, sampling budgets, and optimization stability are jointly constrained. In such settings, conventional sequence-level policy gradients tend to propagate weak and noisy learning signals across long reasoning trajectories. By strengthening the correlation between policy updates and reward-critical generation regions, and by increasing reward density during early training, AF-CuRL substantially improves the learnability of reinforcement learning objectives without relying on additional models or increased computational resources.

### 6.2. Generality of Reinforcement Learning Objective Design

From a methodological perspective, AF-CuRL does not rely on task-specific mathematical semantics or domain-dependent annotations. Its core design principles are motivated by optimization challenges that commonly arise in long-form generation, namely imbalanced credit assignment and reward sparsity under limited training resources. As a result, the proposed framework is potentially applicable to other tasks that require long-context generation and well-defined terminal decision criteria, such as structured problem solving or constrained decision generation.

That said, the applicability of AF-CuRL depends on several assumptions. In particular, the task should admit an identifiable terminal output region, and reliable sequence-level rewards should be available through rule-based evaluation or deterministic graders. In tasks where terminal structure is ambiguous or where reward definitions are inherently subjective, effectively identifying answer regions and designing appropriate curriculum rewards remains an open challenge and requires further investigation. These observations motivate a discussion on how stable reinforcement learning for small language models can benefit practical systems operating under sensing and deployment constraints, which we discuss next.

In this work, mathematical reasoning tasks are used as a controlled and reproducible evaluation setting, where the answer region can be reliably identified using explicit structural markers such as \boxed{}. Importantly, AF-CuRL does not depend on mathematical semantics, but on the existence of an identifiable terminal output region within long-form generation. For tasks without explicit answer markers, similar designs could rely on heuristic delimiters, regions adjacent to the end-of-sequence token, or rule-based terminal detectors, which we leave for future work.

### 6.3. Implications for Sensor-Driven and Edge-Intelligent Systems

Recent advances in intelligent sensing systems increasingly explore the use of large language models as high-level reasoning components, responsible for interpreting structured observations, coordinating multi-step decision processes, and producing explicit terminal outputs. Compared with traditional perception pipelines, such LLM-based reasoning modules offer greater flexibility in handling heterogeneous sensor inputs, symbolic constraints, and rule-based decision logic. However, deploying such models in practical sensing environments introduces strict constraints on model size, memory footprint, and training stability, particularly in edge and embedded scenarios where large-scale models and extensive computational resources are infeasible.

From this perspective, the low-parameter setting studied in this work reflects a realistic operational regime for sensor-driven intelligent systems. Small and medium-scale language models are more compatible with on-device deployment requirements, but they are also more vulnerable to instability during reinforcement learning, especially for long-horizon reasoning tasks. As demonstrated in our experiments, such models tend to exhibit non-terminating or excessively verbose generation behavior when optimized using standard sequence-level reinforcement learning objectives, which is undesirable for time- and resource-sensitive sensing applications.

The proposed AF-CuRL framework addresses this limitation by improving reinforcement learning stability through objective-level design rather than increased model capacity. By concentrating policy updates on answer-critical regions and introducing a curriculum reward schedule that prioritizes stable termination behavior, AF-CuRL enables small language models to learn reliable long-form decision patterns under constrained training budgets. Importantly, this design does not rely on additional critic networks, learned reward models, or extensive sampling, making it particularly suitable for resource-limited sensing systems where simplicity and reproducibility are essential.

Although mathematical reasoning is used as the evaluation benchmark in this study, the underlying challenges—sparse terminal rewards, imbalanced credit assignment, and the need for explicit and well-defined termination—are not specific to mathematics. Similar characteristics arise in sensor-driven decision scenarios such as symbolic condition assessment, anomaly interpretation, and rule-based response generation, where reasoning processes must converge efficiently to a final decision. In such settings, improving termination reliability and generation efficiency can be as critical as maximizing task accuracy.

Overall, the results suggest that, for sensor-driven and edge-intelligent systems employing LLM-based reasoning modules, structured reinforcement learning objectives may play a more decisive role than model scaling in achieving stable and deployable behavior. AF-CuRL provides a practical example of how lightweight reinforcement learning design can enhance the usability of small language models in constrained intelligent systems, offering insights for future research on resource-efficient reasoning in sensing applications.

### 6.4. Limitations

Despite its effectiveness under low-resource conditions, AF-CuRL has several limitations that are important to acknowledge. A central assumption of the proposed method is the existence of an identifiable terminal region within the generated sequence. While this assumption is natural for mathematical reasoning tasks with explicit answer formats, it does not directly extend to open-ended generation problems or tasks where multiple outputs may be equally valid. In such cases, defining answer-critical regions without introducing task-specific heuristics remains nontrivial.

In addition, the empirical evaluation in this work focuses on low-parameter language models trained under constrained computational budgets. Although this setting is intentionally chosen to highlight stability and learnability issues in low-resource reinforcement learning, it remains unclear to what extent the proposed objective design is necessary or beneficial when model capacity and training resources are substantially increased. A systematic investigation across model scales would be required to clarify this point.

Finally, AF-CuRL is designed to improve the stability of reinforcement learning training rather than to expand the intrinsic reasoning capabilities of a language model. When the underlying model lacks sufficient foundational competence, structured objective design alone cannot compensate for limitations in pretraining or supervised data quality. In practice, AF-CuRL should therefore be viewed as complementary to, rather than a replacement for, capability-enhancing approaches such as larger-scale pretraining or improved supervised fine-tuning.

## 7. Conclusions

This work investigates reinforcement learning for long-form generation in low-parameter large language models under constrained computational resources. We show that standard sequence-level policy gradient methods often fail in this regime due to severe reward sparsity and imbalanced credit assignment, leading to unstable and non-convergent generation behavior. To address these challenges, we propose AF-CuRL, a lightweight reinforcement learning framework that improves training stability through structured objective design. By combining answer-focused token reweighting with a two-phase curriculum reward schedule, AF-CuRL concentrates optimization on reward-critical generation regions while providing denser and more learnable training signals during early optimization. Experiments on multiple mathematical reasoning benchmarks demonstrate that AF-CuRL consistently improves both correctness and generation regularity for a 1.5B-parameter model trained under strictly low-resource conditions. These results suggest that, for long-form generation in resource-constrained settings, failures of reinforcement learning are often driven less by model capacity than by objective mismatch, and that carefully designed optimization objectives can substantially improve training stability without increasing model size or computational cost.

## Figures and Tables

**Table 1:** Main results on mathematical reasoning benchmarks. All results are reported on test sets. All methods use comparable 1.5B-scale models and identical evaluation protocols.

	GSM8K	MATH-500	Minerva	Olympiad-Bench	AMC23
DeepSeek-R1-Distill-Qwen-1.5B (Base)	0.43	0.276	0.081	0.062	0.15
OpenMath-Nemotron-1.5B	0.326	0.256	0.074	0.056	0.075
AF-CuRL	0.738	0.566	0.151	0.168	0.275

**Table 2:** Ablation study of AF-CuRL components on GSM8K-dev and MATH-dev. All settings use the same base model and training budget. “Answer-only” disables curriculum reward, while “Curriculum-only” disables answer-focused reweighting.

Model	GSM8K-Dev Acc	MATH-Dev Acc	Boxed (GSM8K/MATH)	Len (GSM8K/MATH)	Notes
DeepSeek-R1-Distill-Qwen-1.5B (Base)	0.42	0.24	0.187/0.2	1599.4/1333	no RL
AF-CuRL (Plain-RL)	0.62	0.47	0.593/0.54	1080.5/1041	no Answer/no Cur
AF-CuRL (Answer-only)	0.65	0.46	0.653/0.473	1244/1038.5	reweight only
AF-CuRL (Curriculum-only)	0.62	0.49	0.62/0.533	1001.3/946.7	curriculum only
AF-CuRL	0.75	0.49	0.8/0.627	824.8/916.8	reweight + curriculum

## Data Availability

Publicly available datasets were analyzed in this study. These datasets can be found at the following repositories on Hugging Face: GSM8K (https://huggingface.co/datasets/openai/gsm8k accessed on 15 January 2026), MATH-500 (https://huggingface.co/datasets/HuggingFaceH4/MATH-500 accessed on 15 January 2026), AIME24 (https://huggingface.co/datasets/HuggingFaceH4/aime_2024 accessed on 15 January 2026), AIME25 (https://huggingface.co/datasets/math-ai/aime25 accessed on 15 January 2026), AMC23 (https://huggingface.co/datasets/zwhe99/amc23 accessed on 15 January 2026), BRUMO25 (https://huggingface.co/datasets/MathArena/brumo_2025 accessed on 15 January 2026), CMIMC25 (https://huggingface.co/datasets/MathArena/cmimc_2025 accessed on 15 January 2026), HMMT25 (https://huggingface.co/datasets/FlagEval/HMMT_2025 accessed on 15 January 2026), Minerva (https://huggingface.co/datasets/math-ai/minervamath accessed on 15 January 2026), and Olympiad-Bench (https://huggingface.co/datasets/Hothan/OlympiadBench accessed on 15 January 2026).The reinforcement learning training data were derived from publicly available benchmark datasets as described above.The codebase used for training and evaluation will be publicly released at: https://github.com/YanZiQinKevin/lzu-deepseek-rl-lab (accessed on 15 January 2026).

## Appendix A. Training Configuration and Implementation Details

This appendix provides detailed training configurations and implementation settings for AF-CuRL to support reproducibility. Unless otherwise specified, all experiments reported in the main text follow the default settings described here.

### Appendix A.1. Model and Data Setup

We conduct all reinforcement learning experiments with DeepSeek-R1-Distill-Qwen-1.5B as the base policy model. Unless otherwise stated, we use the model’s default tokenizer and keep the prompt template and decoding constraints fixed across methods to ensure comparability.

Training and evaluation are implemented with the Hugging Face ecosystem. The full codebase (training, evaluation, and preprocessing) will be open-code.

### Appendix A.2. Reinforcement Learning Training Procedure

Reinforcement learning is performed using sequence-level policy gradient optimization with a total of 600 training steps. In accordance with the curriculum reward schedule, training is divided into two phases:

- Phase 1: The first 100 steps use only dense intermediate reward signals.
- Phase 2: The remaining steps gradually incorporate correctness-oriented rewards.

Model checkpoints are saved every 200 training steps. Evaluation on the validation set is performed every 100 steps to monitor training stability and convergence.

### Appendix A.3. Generation and Sampling Settings

The maximum input prompt length is set to 384 tokens, and the maximum generation length is set to 512 tokens. Generation uses temperature sampling with temperature = 0.7 and top-p = 0.9.

Single-sample generation (K = 1) is used by default during both training and validation to maintain consistency across experimental settings.

### Appendix A.4. Optimizer and Parameter Updates

Parameter updates are performed using the AdamW optimizer with a learning rate of 2 × 10^−5^ and weight decay set to 0. The optimizer hyperparameters are $\beta_{1}=0.9$, $\beta_{2}=0.9$ and $\epsilon =10^{-8}$.

Gradient norm clipping is applied with a maximum norm of 1.0. All experiments are conducted with a fixed random seed (seed = 42).

### Appendix A.5. Parameter-Efficient Fine-Tuning (LoRA)

Parameter-efficient fine-tuning is implemented using LoRA. The specific configuration is as follows:

- LoRA rank r = 8
- LoRA scaling factor α = 16
- Dropout rate = 0.05

Only LoRA parameters are updated during training, while all base model weights remain frozen.

The LoRA configuration (r = 8, α = 16) is selected as a practical compromise under low-resource training conditions. Smaller ranks may limit adaptation capacity, while larger ranks increase trainable parameters and memory overhead, potentially reducing training stability on constrained hardware. This configuration follows common practice in parameter-efficient fine-tuning and is kept fixed across all experiments to ensure reproducibility and fair comparison.

### Appendix A.6. Answer-Focused Token Weighting

In AF-CuRL, different token regions within the generated sequence are assigned different weights. Tokens belonging to the final answer region are assigned a weight of 1.0, while tokens in the reasoning region are assigned a weight of 0.2. This weighting is applied during policy optimization as described in Section 3.3.

### Appendix A.7. Reward Baseline and Smoothing

An exponential moving average (EMA) reward baseline is used to reduce variance in policy gradient updates. The EMA smoothing coefficient is set to 0.05. Unless otherwise specified, this baseline mechanism is enabled in all experiments.

### Appendix A.8. Validation Monitoring

During training, periodic validation is performed to monitor model behavior. At each validation step, up to 15 samples are randomly selected and evaluated using single-sample generation. Validation is conducted every 100 training steps.

### Appendix A.9. Computational Environment

All experiments are executed on CUDA-enabled GPU environments. No system-level optimizations specific to individual experiments are applied beyond standard deep learning frameworks.

## Appendix B. Quantitative Results

**Table A1:** Reports correctness and behavior-related metrics for the default AF-CuRL configuration and the extended Phase-1 variant under identical evaluation settings.

	Acc	Boxed_Rate	Avg_Len
base_distill_gsm_math_dev_k2	0.333	0.153	1497.1
base_distill_gsm8k_dev_k2	0.42	0.187	1599.4
base_distill_math_dev_k2	0.24	0.2	1333.0
base_distill_math_test_k2	0.287	0.187	1399.6
base_distill_gsm8k_test_k2	0.433	0.193	1638.1
			
afcurl_step100_gsm_math_dev_k2	0.637	0.733	882.2
afcurl_step100_gsm8k_dev_k2	0.747	0.8	824.8
afcurl_step100_math_dev_k2	0.493	0.627	916.8
afcurl_step100_math_test_k2	0.58	0.7	873.3
afcurl_step100_gsm8k_test_k2	0.713	0.773	828.5
			
afcurl_step200_gsm_math_dev_k2	0.59	0.633	994.9
afcurl_step200_gsm8k_dev_k2	0.733	0.74	947.9
afcurl_step200_math_dev_k2	0.473	0.573	999.9
afcurl_step200_math_test_k2	0.587	0.64	962.4
afcurl_step200_gsm8k_test_k2	0.76	0.753	857.5

## Appendix C. Failure Mode Analysis of the Base Model

This appendix reports descriptive statistics of generation behavior produced by the base model across multiple mathematical reasoning benchmarks, without reference to reinforcement learning methods.

### Appendix C.1. Behavioral Metrics and Data Source

We analyze generation logs produced by the base model under a unified evaluation setup. The reported metrics include boxed rate, average output length, and related length statistics computed directly from model outputs. Table A2 summarizes these metrics across benchmarks.

To characterize generation behavior during reasoning, we focus on the following metrics:

Boxed rate: the proportion of outputs that contain a properly formatted final answer marker (e.g., \boxed{}), indicating whether the model produces an explicit and verifiable conclusion at the end of reasoning.

Average output length (avglen): the average length of generated sequences, reflecting reasoning efficiency and convergence.

**Table A2:** Behavioral statistics of DeepSeek-R1-Distill-Qwen-1.5B across math benchmarks.

Task_Name	Num_Samples	Num_Correct	Acc@1	Has_Valid_Boxed_Rate	Boxed_Rate_in_Correct	Boxed_Rate_in_Incorrect	Avg_Char_Len	Avg_Char_Len_Correct	Avg_Char_Len_Incorrect	Avg_Think_Char_Len
AIME24	30.00	3.00	0.10	0.07	0.33	0.04	6673.07	6111.00	6735.52	6023.00
AIME25	30.00	1.00	0.03	0.00	0.00	0.00	6914.07	6133.00	6941.00	6263.13
AMC23	40.00	15.00	0.38	0.43	0.87	0.16	5177.33	4255.40	5730.48	4384.30
BRUMO25	30.00	4.00	0.13	0.07	0.50	0.00	6603.97	6718.75	6586.31	6108.80
CMIMC25	40.00	1.00	0.03	0.05	1.00	0.03	6686.80	3346.00	6772.46	6100.10
GSM8K	500.00	384.00	0.77	1.00	1.00	0.98	1666.56	1610.61	1851.77	561.08
HMMT25	30.00	0.00	0.00	0.00	0.00	0.00	7238.87	0.00	7238.87	6736.33
MATH-500	500.00	303.00	0.61	0.64	0.90	0.23	3980.33	3066.41	5386.02	3093.47
Minerva	272.00	49.00	0.18	0.57	0.96	0.48	4922.32	4563.35	5001.20	3895.15
Olympiad-Bench	674.00	147.00	0.22	0.30	0.89	0.13	5757.78	3926.89	6268.48	5000.61

### Appendix C.2. Non-Convergent Outputs and Missing Termination Signals

Table A2 shows that the base model frequently fails to produce explicitly marked final answers on several benchmarks. In these cases, generated outputs often terminate without a valid answer marker or remain in an intermediate reasoning state.

### Appendix C.3. Divergent Reasoning and Redundant Generation

Beyond output regularity, the base model also exhibits substantial redundancy in generation. The average output length is consistently high across benchmarks, with especially long sequences produced on more difficult tasks.

### Appendix C.4. Formatting Instability and Answer Extraction Failures

In some cases, even when the generated output contains numerically relevant information related to a correct solution, the formatting remains unstable. Examples include inconsistent answer placement, mixed symbolic representations, or the absence of explicit answer delimiters. Such formatting inconsistencies make automated answer extraction more difficult.
